# CEREBRAL ATROPHY CORRESPONDING TO TOOTH LOSS IN ELDERLY INDIVIDUALS

**DOI:** 10.1093/geroni/igz038.3296

**Published:** 2019-11-08

**Authors:** Yojiro Umezaki, Rui Egashira, Michiko Makino, Toru Naito

**Affiliations:** 1 Department of General Dentistry, Fukuoka Dental College, Fukuoka, Japan; 2 Fukuoka dental college, Fukuoka, Japan

## Abstract

Recent advances in the aging of Japanese society, have meant that the chance of encountering dementia patients in dental clinics is dramatically increasing. Many studies have shown that the brain volume decreases along with the progression of dementia. Although previous studies have reported a relationship between tooth loss or periodontitis and the onset of dementia, the pathological mechanisms underlying this association have not been elucidated. In this study, we focused on the relationship between the oral condition and brain atrophy to discuss how to adequately deal with dementia patients. This study included fifteen participants who underwent brain MRI (magnetic resonance imaging). We obtained information on the oral condition, lifestyle, cognitive function and brain atrophy. The cognitive function was assessed using the Mini-Mental State Examination (MMSE). MR images of each patient were analyzed with the Voxel-based Specific Regional Analysis System for Alzheimer's Disease to quantitate the degree of brain atrophy. The study population included 4 male and 11 female patients. The mean age was 75.9 years. The mean number of present teeth was 15.0. The median MMSE score was 26. The degree of atrophy of the whole brain was significantly correlated with the number of present teeth (r=-0.72, p<0.05) and the presence of a daily exercise habit (r=-0.66, p<0.05). This study showed that the number of present teeth could be an indicator reflecting the progress of dementia. Preserving the teeth as well as the acquisition of a regular exercise habit might be important for preventing dementia.

